# The Presidency of the Royal College of Physicians

**Published:** 1896-03-21

**Authors:** 


### The Hospital

An Institutional Journal of

The Medical Sciences and Hospital Administration, with

AN EXTRA NURSING SUPPLEMENT.

Vol. XIX.—No. 495.] [March 21, 1896.

### THE PRESIDENCY OF THE ROYAL COLLEGE OF PHYSICIANS.

We hasten to correct a statement which appeared in the British Medical Journal last week to the effect that Dr. Samuel Wilks is not now disposed to accept the Presidency of the Royal College of Physicians. The matter is most urgent, for the election must take place on March 30th, and the continuance for a week of such a report uncontradicted is likely to be most prejudicial to what we consider a step of the greatest importance, both to the profession and the college, viz., the election of Dr. Wilks to a post to which he has every claim.

We have the highest authority for saying that, so far from it being correct to say that Dr. Wilks is not now disposed to accept office, he is quite prepared to do so and to undertake the duties which pertain to it.

We need not say how extremely appropriate it would be at the present juncture that this honour should be conferred upon him. When in 1893 the sudden and unexpected death of Sir Andrew Clark made it necessary to find a new President, the voting was almost equally divided between Sir Russell Reynolds and Dr. Wilks, the former obtaining seventy-five and the latter seventy-two votes. Sir Russell Reynolds thus only obtained the presidency by a majority of three, and it must not be forgotten that in the first ballot Dr. Wilks actually headed the poll, the votes in his favour being forty-five against forty-three for Sir Russell Reynolds. There can then be no possible doubt, so far as the College is concerned, not only that the appointment of Dr. Wilks would be considered fitting and appropriate, but that he would be personally acceptable to his colleagues as their president. So far as the opinions of those outside the walls of the College are of importance in the matter it is also quite certain that of all the names suggested, not only is that of Dr. Wilks by far the best known, but that it is one which is peculiarly associated in the mind of the profession with high ideals. His constant devotion to medical science, his unswerving rectitude, and his unflinching loyalty to his professional brethren, are so well recognised that the whole rank and file of medicine would be glad to know that the name of Samuel Wilks was to be enrolled on the honourable list of presidents of the Royal College of Physicians. There is some reason for thinking that, by devoting his best years to the scientific investigation of those aspects of disease which come least before the eye of the people, and by his habit of looking on wealth, position, and those honours which some consider the great prizes of life, as of but secondary importance in comparison with increase of knowledge, dissipation of error, and the confusion of al quackery, Dr. Wilks may have failed to shine before men and impress the outer world to the same degree that many lesser lights have done. All the more necessary is it then for us to say, as we do with full knowledge, that Dr. Wilks is a man whom the medical profession delights to honour and will be still more delighted to see honoured.

This brings us back to an important point. It is most unfortunate that any doubt should have been thrown upon the willingness of Dr. Wilks to accept office, for in every way—in other ways besides those we have mentioned—he is exactly the man that is wanted at the present moment.

There is no reason why we should hide the fact that the conditions just now are peculiar. The custom has held sway for long that the President should retain office for a series of years, generally about five; and no one can doubt that the honours so usually conferred by the Crown on the holders of this high office have a certain relation to the fact that such honours do not require to be repeated at very short intervals. Sir Russell Reynolds, however, has, to the regret of everyone, found it necessary to decline re-election after a very short period of office, and if it is desired to maintain the tradition which associates the Presidency with some mark of Royal favour, it is of the greatest importance that whoever is appointed as his successor should be one whom Royalty as well as his profession will be glad to honour. Now in this matter the circumstances are peculiar and worthy of the most careful consideration of the Royal College, for by the position occupied by Dr. Wilks in connection with the raising of a large sum of money for the re-endowment of Guy's Hospital, he is practically the colleague of the Prince of Wales in what will probably turn out to be such a display of public charity as may fittingly adorn the close of a century which has not been backwards in good works. The interest that Her Majesty the Queen has always shown in the progress of medicine is well known; and still better known to those who are cognisant of hospital affairs, is the active personal interest taken by the Prince of Wales and by several members of his family in all the details of hospital administration.

The Prince of Wales has lately shown much concern in regard to the financial condition of Guy's Hospital, for which £15,000 a year is now wanted to re-establish it in the position of usefulness which it formerly occupied, and we have reason to believe that, as the result of movements—separate perhaps, but still associated—which depend, on the one hand, on those social influences which emanate from Marlborough House, and, on the other, on the willingness of "Guy's men" all the world over to rally round Dr. Wilks for the good of their old hospital, will result in such a display of public munificence as will astonish those who profess to think that charity is dead amongst us.

If there has been one characteristic in Dr. Wilks' career more prominent than his loyalty to science, it has been his loyalty to "Guy's." Royalty and science are then in this case co-operating in the cause of charity, and we believe that if the Royal College of Physicians should appoint Dr. Wilks their President, the Crown, which must always be the true fount of honour, will not be backward in bestowing such marks of royal favour as shall demonstrate once again the sympathy and approval which is felt on the throne for those whose life is spent in the advancement of science and in the good of humanity.

